# Pedal access for genicular artery embolization: technique description and procedural workflow

**DOI:** 10.1093/jscr/rjag439

**Published:** 2026-06-10

**Authors:** Mohmmed Margni, Babatunde Almaroof, Fayad Mohamed, Ahmed Rafei

**Affiliations:** Department of Vascular Surgery, Vascular Institute of Michigan, 1325 South Linden Road, Flint, MI 48532, United States; Department of Vascular Surgery, Vascular Institute of Michigan, 1325 South Linden Road, Flint, MI 48532, United States; Department of Vascular Surgery, Vascular Institute of Michigan, 1325 South Linden Road, Flint, MI 48532, United States; Department of Vascular Surgery, Vascular Institute of Michigan, 1325 South Linden Road, Flint, MI 48532, United States

**Keywords:** genicular artery embolization, pedal arterial access, tibial access, knee osteoarthritis, endovascular technique

## Abstract

Genicular artery embolization has emerged as a minimally invasive treatment for moderate-to-severe knee osteoarthritis and is traditionally performed via femoral access. With increasing operator familiarity with distal arterial techniques, pedal access represents a feasible alternative. We describe a single-operator technique for performing genicular artery embolization via pedal arterial access developed over more than a decade of experience. Pre-procedural assessment includes duplex ultrasound to evaluate vessel patency and diameter. Under ultrasound guidance, the dorsalis pedis or distal posterior tibial artery is accessed, followed by sheath placement and systemic anticoagulation. Retrograde angiography and roadmap guidance facilitate selective catheterization of genicular branches using a mini-RIM catheter and a no-wire “Jabada” technique. Embolization is performed to reduce hypervascularity while preserving flow. This approach allows efficient workflow, reduced radiation and contrast use, and early ambulation, providing a practical alternative to femoral access for genicular interventions.

## Introduction

Genicular artery embolization (GAE) was first reported for osteoarthritis in 2015 by Okuno in Japan [1]. It has since emerged as a minimally invasive procedure for moderate-to-severe osteoarthritis [2]. GAE has traditionally been performed via ipsilateral or contralateral femoral access, reflecting operator familiarity [2]. With the advent of pedal tibial artery access in peripheral artery disease (PAD) treatment, vascular operators have become comfortable using this approach for lower extremity interventions [3]. In our practice, we have almost exclusively adopted the pedal approach as the initial access for GAE. From our experience, pedal access affords quicker recovery with less radiation and contrast use. We describe our technique for pedal access for GAE.

## Pre-procedural assessment

The pedal approach for revascularization is our primary route [4] unless workup shows severe pan-tibial and pedal occlusive disease (“desert foot”), local infection, open wounds compromising a sterile field, or extreme foot tremors prohibiting safe arterial access [5, 6]. When GAE was introduced to our practice, pedal access was a logical alternative to retrograde or antegrade femoral access due to lower morbidity and no post-procedural restrictions [7]. This is a single-operator technique with >13 years of pedal-access experience. All patients undergo arterial duplex ultrasound of the ipsilateral limb to assess occlusive disease and measure the diameters of the dorsalis pedis and distal posterior tibial arteries before bifurcation.

## Pedal arterial access

**Figure 1:**
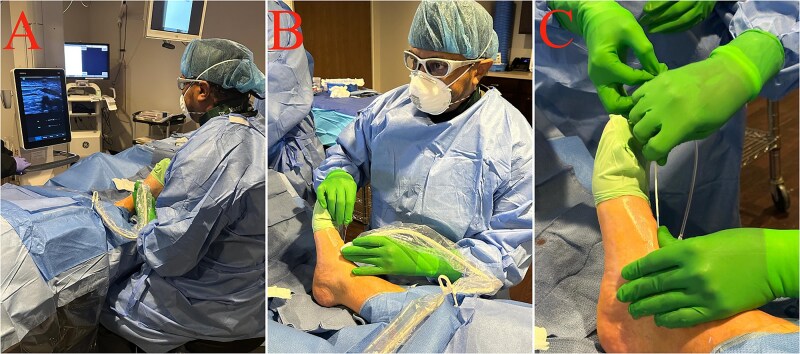
Ultrasound-guided pedal arterial access. (A) Procedural setup with the patient supine and the ipsilateral foot prepped and draped; the ultrasound system is positioned to allow real-time guidance. (B) Ultrasound-guided puncture of a pedal artery on the dorsum of the foot using a sterile probe cover and oblique needle approach with continuous needle-tip visualization. (C) Guidewire advancement through the access needle following successful puncture (micropuncture technique).

Under direct ultrasound guidance, the pedal artery (dorsalis pedis or distal posterior tibial) is accessed at a 45° angle after infiltration with 1% lidocaine (Fig. 1A–C). The needle tip must remain continuously visible to ensure true-lumen entry without wall contact. Because pedal vessels are smaller than the femoral artery, dissection is more likely if this step is overlooked [8]. A 5F Terumo pedal sheath is inserted using the supplied 0.16 wire advanced gently through the access needle. Tactile feedback is critical; there should be no resistance. If resistance is encountered, the wire is withdrawn, back-bleeding is reassessed through the needle, and the needle tip is visualized within the artery. For distal posterior tibial access, tortuosity may require foot flexion to straighten the distal segment. A floppy J-shaped 0.14 wire can help.

## Sheath confirmation and anticoagulation

**Figure 2:**
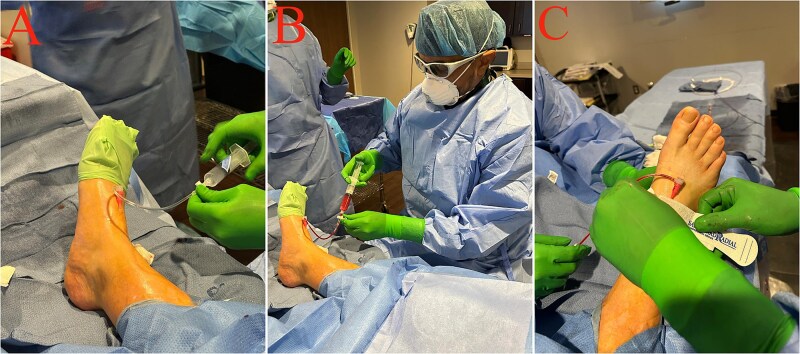
Pedal sheath setup, confirmation, and securement. (A) Short pedal sheath secured on the dorsum of the foot and connected to extension tubing and a three-way stopcock. (B) Aspiration/back-bleeding through the sheath/extension tubing confirming intraluminal placement prior to angiography. (C) Application of a hemostatic compression device to stabilize the access region and support hemostasis while the sheath remains in place.

After sheath placement, blood return on aspiration is confirmed (Fig. 2B). If return is not robust, tibial angiography is performed to evaluate occlusion or dissection. If satisfactory, the sheath is flushed with heparinized saline and intravenous heparin is administered: 4000 units for patients <70 kg and 5000 units for patients ≥70 kg (Fig. 2A).

## C-arm positioning and initial angiography

**Figure 3:**
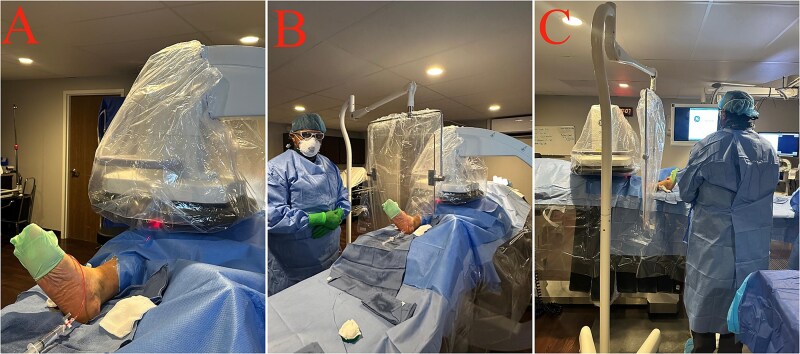
Fluoroscopy setup during the procedure after pedal access. (A) Covered C-arm positioned over the draped foot following pedal access, with extension tubing maintained for flushes/contrast injection. (B) Wide view of the procedure-room setup showing the patient under sterile drapes, the covered C-arm, and protective shielding in place during fluoroscopy. (C) Operator working at the sterile field while viewing imaging monitors during the fluoroscopic phase of the procedure.

**Figure 4:**
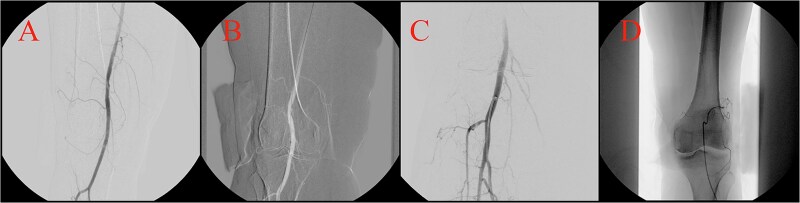
Angiographic navigation from pedal access to selective genicular catheterization. (A) Initial retrograde angiogram obtained through the pedal sheath demonstrating the arterial roadmap used for subsequent catheter navigation. (B) Roadmap-guided wire advancement from pedal access toward the distal superficial femoral/popliteal segment. (C) Mini-RIM catheter positioned after advancement from the pedal approach, providing stable support for selective branch cannulation. (D) Distal microcatheter position within the target genicular artery prior to selective angiography and embolization.

After sheath placement, confirmation of back-bleeding and heparin administration, the C-arm is positioned to cover the lower third of the thigh and a few inches below the knee joint line, encompassing the genicular arteries in most patients (Fig. 3A). Using a 10 ml syringe containing 3 ml of Visipaque mixed with 7 ml of heparinized saline, a retrograde angiogram is obtained via the sheath before introducing wires or catheters (Fig. 4A). This usually visualizes up to the descending genicular artery, except in patients with ectatic or severely stenotic popliteal and superficial femoral arteries. After a satisfactory angiogram, the roadmap technique is used to reduce contrast use and radiation exposure. Protective shielding is used, and the operator views fluoroscopy monitors during roadmap-guided branch selection (Fig. 3B and C).

## Catheter strategy and branch cannulation

Using the roadmap, a 0.35 hydrophilic wire selects the distal superficial femoral artery (Fig. 4B), followed by advancement of a 5F mini–Rösch inferior mesenteric (RIM) catheter (Fig. 4C). Compared with the conventional RIM catheter, the mini-RIM can form an inverted U-shape within smaller superficial femoral and popliteal arteries, whereas the conventional RIM generally requires larger-caliber vessels. Once the mini-RIM forms its shape, its end hole faces caudally and downward, becoming parallel to the descending genicular artery course. The wire is then removed. With roadmap guidance, we use a no-wire selection method called the “Jabada” technique, named after a Sudanese fruit-harvesting tool, to select and cannulate genicular branches (Supplementary Video S1). A 2.4F microcatheter is then advanced distally into each branch (Fig. 4D).

## Angiography and embolization workflow

**Figure 5:**
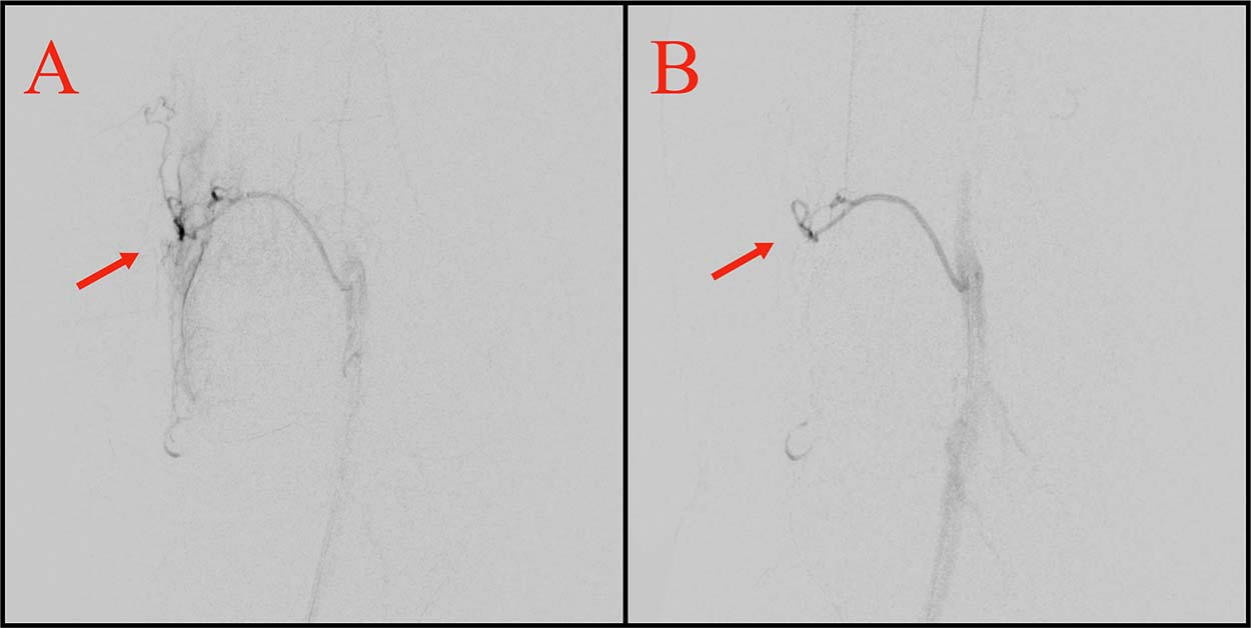
Representative selective genicular angiograms before and after embolization. (A) Pre-embolization angiogram of the superior lateral genicular territory demonstrating abnormal hypervascularity/blush (arrow). (B) Post-embolization angiogram from a representative treated genicular branch showing marked reduction/resolution of the previously identified hypervascularity (arrow), with preservation of arterial patency.

After a genicular branch is selected to its distal segment using the roadmap, the roadmap is removed and a small dose of nitroglycerin is injected into the distal genicular artery. Angiography is then performed using a 3 ml syringe containing 1 ml of Visipaque mixed with 2 ml of heparinized saline (Fig. 5A). Embolization is then performed. If permanent embolic material is used, the goal is elimination of hypervascularity while maintaining flow; if temporary agents are used, the goal is transient stasis. A post-embolization angiogram is obtained for each branch using the same one-third contrast mixture (Fig. 5B). We begin with the most proximal genicular branch and proceed distally; the recurrent genicular artery is treated last. This sequence reduces wire exchanges, minimizes radiation exposure, and improves efficiency. We focus on the most symptomatic region but attempt to cannulate all genicular arteries and treat those with significant hypervascularity. If significant proximal tortuosity, severe ostial stenosis, or unfavorable angulation is present, a soft-tip shapeable 0.14 wire is used through the mini-RIM to select that artery, followed by advancement of the microcatheter over the wire.

## Completion and device removal

After completing GAE, the mini-RIM is straightened before removal using the original 0.35 wire. This can be done by selecting a branch with the wire or by positioning the wire in the distal popliteal artery via the other tibial artery. The catheter is removed smoothly over the wire, followed by wire removal.

## Hemostasis and post-procedural care

After wire removal, the sheath is withdrawn and flushed with heparinized saline. Hemostasis is achieved manually or mechanically. We prefer the SafeGuard device for most pedal-access cases because it allows reinflation of the compression balloon if needed after deflation (Fig. 2C). One hour after the procedure, the patient ambulates in the recovery room and may use the bathroom. The access site is reassessed; if dry, the balloon device is removed, and a 4 × 4 gauze dressing is applied. The patient is instructed to remove the dressing the next day.

## Access-site complications and comparison with femoral access

Compared with femoral access, pedal access may reduce groin-related complications, including hematoma, pseudoaneurysm, arteriovenous fistula, bleeding, and prolonged immobilization [9]. In our experience, it has allowed early ambulation and fewer access-site restrictions. However, pedal access has specific risks related to smaller vessel caliber, including vasospasm, dissection, thrombosis or occlusion, hematoma, perforation, distal embolization, and failed access in diseased or tortuous vessels [7, 10]. Careful duplex assessment, ultrasound-guided puncture, gentle wire manipulation, anticoagulation, and meticulous hemostasis help minimize these risks. Formal comparison of complications between pedal and femoral access was beyond the scope of this technical report, and future comparative studies are needed.

## Supplementary Material

Jabada_for_GAE_rjag439

## References

[1] Okuno Y, Korchi AM, Shinjo T et al. Transcatheter arterial embolization as a treatment for medial knee pain in patients with mild to moderate osteoarthritis. Cardiovasc Intervent Radiol 2015;38:336–43. 10.1007/s00270-014-0944-8 24993956

[2] Tyagi R, Ahmed SS, Koethe Y et al. Genicular artery embolization for primary knee osteoarthritis. Semin Intervent Radiol 2022;39:125–9. 10.1055/s-0042-1745798 35782001 PMC9246485

[3] McGuirl D, Giles KA. Retrograde tibio-pedal access for endovascular interventions for treating peripheral arterial disease. Ann Vasc Surg 2024;107:136–9. 10.1016/j.avsg.2023.12.105 38582196

[4] Margni M, Almaroof B, Mohamed F et al. The use of the ipsilateral dorsalis pedis artery approach for transarterial embolization in symptomatic chronic plantar fasciitis: a case report. *J Surg Case Rep* 2025;2025:rjaf1018. 10.1093/jscr/rjaf1018 PMC12722164 41446003

[5] Williams C, Pasrija D, Pierre L et al. Arterial lines. [Updated 2025 Mar 23], StatPearls [Internet]. Treasure Island (FL): StatPearls Publishing, 2025. Available from: https://www.ncbi.nlm.nih.gov/books/NBK499989/. 29763165

[6] Gornik HL, Aronow HD, Goodney PP et al. 2024 ACC/AHA/AACVPR/APMA/ABC/SCAI/SVM/SVN/SVS/SIR/VESS guideline for the management of lower extremity peripheral artery disease: a report of the American College of Cardiology/American Heart Association Joint Committee on Clinical Practice Guidelines. Circulation 2024;149:e1313–410. 10.1161/CIR.0000000000001251 38743805 PMC12782132

[7] Sajan A, Lerner J, Kasimcan MO et al. Feasibility and technique of retrograde pedal access for genicular artery embolization. J Vasc Interv Radiol 2023;34:2030–3. 10.1016/j.jvir.2023.07.012 37495097

[8] Lorbeer R, Grotz A, Dörr M et al. Reference values of vessel diameters, stenosis prevalence, and arterial variations of the lower limb arteries in a male population sample using contrast-enhanced MR angiography. PloS One 2018;13:e0197559. 10.1371/journal.pone.0197559 29924802 PMC6010244

[9] Ciprian Cacuci A, Krankenberg H, Ingwersen M et al. Access site complications of peripheral endovascular procedures: a large, prospective registry on predictors and consequences. J Endovasc Ther 2021;28:746–54. 10.1177/15266028211025044 34137662

[10] Giannopoulos S, Palena LM, Armstrong EJ. Technical success and complication rates of retrograde arterial access for endovascular therapy for critical limb ischaemia: a systematic review and meta-analysis. Eur J Vasc Endovasc Surg 2021;61:270–9. 10.1016/j.ejvs.2020.11.020 33358346

